# Routine Versus Clinically Indicated Cerebrospinal Fluid Sampling During Diversion Procedures: A Second-Cycle Audit at a Tertiary Neurosurgical Centre in the United Kingdom

**DOI:** 10.7759/cureus.119208

**Published:** 2026-11-05

**Authors:** Yashjeet Basu

**Affiliations:** 1 Neurosurgery, Royal Victoria Infirmary, Newcastle upon Tyne, GBR

**Keywords:** antimicrobial stewardship, cerebrospinal fluid, clinical audit, csf diversion, ventriculoperitoneal shunts

## Abstract

Background

Cerebrospinal fluid (CSF) cultures contribute to the investigation of suspected infection associated with CSF diversion procedures. However, routine intraoperative sampling in patients without suspected infection may yield organisms, often skin commensals, of uncertain or insignificant clinical significance. A first-cycle local audit of 172 samples found that most cultures were negative and few led to treatment, and recommended avoiding routine sampling in elective cases where infection was not suspected. The primary objective of this second-cycle audit was to determine whether routine CSF sampling (performed when infection was not suspected) led to culture-driven changes in management, compared with clinically indicated sampling (performed when infection was suspected). Secondary objectives were to compare culture positivity and antibiotic initiation between the two groups and to assess the consistency of documentation of the sampling indication.

Methods

A retrospective re-audit of all intraoperative CSF specimens from shunt and external ventricular drain procedures at a tertiary neurosurgical centre in the United Kingdom was undertaken (from August 2024 to July 2025). Specimens were classified as routine or clinically indicated using pre-specified criteria applied to operative notes, electronic records, and microbiology request forms. The author classified all specimens and extracted all outcomes; there was no independent second reviewer. The primary outcome was a documented change in management attributable to the culture result. Categorical data were summarised as counts and percentages. No formal statistical testing was undertaken, consistent with the descriptive nature of this audit.

Results

Of 78 specimens, 60 (76.9%) were routine, and 18 (23.1%) were clinically indicated. Culture positivity was 16.7% (10/60) for routine and 44.4% (8/18) for clinically indicated specimens. None of the 10 routine positive cultures led to a change in management (0/10 positive routine specimens), whereas 7 of 8 indicated positive cultures did (87.5%). Microbiology request wording and clinical documentation were discordant on the indication in 14 specimens (17.9%).

Conclusion

Routine intraoperative CSF sampling persisted despite first-cycle guidance and, in this cohort, did not change documented management, whereas clinically indicated sampling accounted for all culture-driven management changes. The small number of events limits precision, and the absence of documented harm in a retrospective audit cannot establish that omitting routine sampling is safe. Therefore, a prospective evaluation of an indication-based pathway, with structured documentation of the indication and the missed-infection rate as a balancing measure, is warranted.

## Introduction

Cerebrospinal fluid (CSF) diversion is among the most frequently performed neurosurgical interventions, and device-related infection remains one of its most burdensome complications. A 2023 systematic review and meta-analysis of internal ventricular shunts in adults (44 studies, 57,259 patients) reported a pooled infection rate of 4.75% (95% confidence interval (CI) 3.80-5.92%) [1], and according to a large meta-analysis cited by the Infectious Diseases Society of America (IDSA), the overall pooled incidence of external ventricular drain (EVD)-related infection was 11.4 per 1,000 catheter-days [2]. Most shunt infections are reported to occur within the first few weeks of surgery, with around 70% diagnosed within one month and 90% occurring within six months [3] and are thought to result from colonisation of the device with skin flora at the time of surgery [2,4]. Coagulase-negative staphylococci, especially *Staphylococcus epidermidis*, account for approximately 31-37% of isolates and *Staphylococcus aureus* for 18-26% [5]. *Cutibacterium acnes* (formerly known as *Propionibacterium acnes*) accounted for 9% of isolates in adults [5]. Gram-negative organisms are less frequent but accounted for 22.9% of isolates in a recent paediatric and young adult cohort [6].

Infection also drives reoperation and resource use. In the UK Shunt Registry (2004-2013), revision rates among adults were 12.8% at 90 days and 17.4% at one year, and infection was one of the main reasons for revision, alongside underdrainage [7]. In the United States, admissions for shunt infection requiring revision had the highest median cost (US$71,300) and the longest median length of stay (25 days) compared with initial shunt placement [8]. This burden creates a diagnostic issue. Skin commensals are simultaneously the most common true pathogens, while also being the most common culture contaminants. Therefore, a positive CSF culture can be difficult to interpret, particularly in a patient who is clinically well [2]. Routine intraoperative sampling performed without clinical suspicion of infection therefore risks generating results of uncertain significance which may then turn into further antibiotic courses, revision procedures, externalisation, and prolonged admissions.

Routine intraoperative CSF sampling during diversion procedures has been a long-standing practice in our unit. A first-cycle local audit of 172 samples (unpublished institutional audit poster: Mohamed S, Cunningham C, Narayanan M, Ross N. Is CSF Sampling Useful During Routine Shunt Surgery? 2025) found that approximately 92% of cultures were negative, 8% were positive, and only about 4% of all samples led to treatment, with most isolates being typical skin commensals. These findings mirror published data. In a paediatric series of 174 ventriculoperitoneal shunt revisions, Steinbok et al. identified 19 cases in which shunt components grew bacteria without CSF growth or clinical evidence of infection; only one was treated, and contamination did not increase the risk of subsequent shunt malfunction [9]. Similarly, Meredith et al. showed that coagulase-negative staphylococci isolated from broth culture alone, without growth on solid media, usually represent contamination [10]. On this basis, the first cycle recommended avoiding routine sampling in elective diversion procedures where infection was not suspected, while retaining clinical judgement for emergency procedures. However, the first cycle did not distinguish routine from clinically indicated specimens, so it could not determine whether the observed treatment decisions arose from routine sampling at all. The second cycle was designed to address this gap.

The wider literature also does not support routine CSF sampling for infection surveillance. A 2026 systematic review of routine versus clinically indicated CSF sampling from EVDs identified 14 studies, of which only two directly compared the two strategies (routine sampling versus clinically indicated sampling), and neither showed a clear advantage for routine sampling [11]. The authors concluded that catheter duration, system manipulation, and patient-related factors drive infection risk more than sampling frequency, and suggested future prospective studies with standardised protocols [11].

In this audit, routine sampling refers to intraoperative CSF sampling performed when infection was not clinically suspected, and clinically indicated sampling refers to sampling performed when infection was suspected based on intraoperative findings or clinical documentation. The primary objective was to determine whether routine sampling led to documented culture-driven changes in management, compared with clinically indicated sampling. The secondary objectives were to compare culture positivity and antibiotic initiation between the two groups and to assess agreement between the documented clinical indication and the wording of the microbiology request.

## Materials and methods

Study design

This was a retrospective, single-centre clinical audit (second cycle) of all intraoperative CSF specimens obtained during CSF diversion procedures at a tertiary neurosurgical centre in the United Kingdom between August 1, 2024 and July 31, 2025. The audit standard, derived from the first cycle and consistent with national and international guidance [2], was that intraoperative CSF should be sent for culture when infection is clinically suspected. The second cycle extended the first by distinguishing routine from clinically indicated sampling, rather than re-measuring an identical standard. All patients were adults aged 18 years or older.

Case identification

Specimens were identified from a laboratory information system extract, provided by the local microbiology department, of every CSF specimen received from the operating theatres during the audit period. No random, consecutive, or convenience sampling was applied, and no specimens were excluded, except those that did not meet the eligibility criteria mentioned below. The extract contained specimen receipt dates, specimen numbers, patient identifiers, specimen site and type, microscopy and culture findings, and the clinical details entered on the request form. Multiple result entries relating to the same specimen were consolidated using the unique specimen number, and direct and enrichment culture results were reviewed together to determine the final culture result. The operative note and electronic patient record were then reviewed for every specimen.

Laboratory methods

CSF specimens were processed by the hospital's accredited clinical microbiology laboratory according to its standard operating procedures. Processing included microscopy with cell count and gram stain, direct culture on solid media and enrichment culture in broth, and incubation. Organism identification and comments on likely clinical significance were provided by the laboratory and reviewing microbiologists. No audit-specific laboratory procedures were introduced.

Eligibility criteria

Eligible specimens were CSF samples obtained during ventriculoperitoneal, lumboperitoneal, subduroperitoneal, or ventriculoatrial shunt insertion, removal, or revision, and EVD insertion, removal, or replacement. Excluded were hardware or device specimens (valves, tubing, reservoirs, catheter tips), non-CSF specimens (e.g., abdominal catheter samples), and specimens labelled "sample", "shunt tubing", or "valve" unless the documentation confirmed that free-flowing CSF was obtained.

Variables collected

Variables collected were procedure type; device; action (insertion, removal, revision, exploration, externalisation); culture result and organism; microbiology comment on likely significance; antibiotics started and their timing relative to the culture result; change in management attributed to the culture result; return to theatre attributable to the result; readmission for infection; re-operation for infection; narrative indication from the operative note or electronic record; and microbiology request wording.

Classification of sampling indication

Specimens were classified according to the documented clinical context before or at the time of sampling. Specimens were classified as routine (infection not suspected) when they arose from elective diversion surgery without clinical suspicion or signs of infection; surgery for hydrocephalus alone (subarachnoid haemorrhage, tumour, idiopathic intracranial hypertension, normal-pressure hydrocephalus) without features of infection; revision, exploration, or valve change for mechanical reasons without concern for infection; or procedures in which the documentation was silent on infection, and there was no fever, raised inflammatory markers, pus, or wound breakdown.

Specimens were classified as clinically indicated (infection suspected) when there was documented suspicion of shunt infection, ventriculitis, or meningitis; exposed or externalised hardware with discharge or erythema; re-presentation with fever, wound breakdown, purulent drainage, or raised inflammatory markers; or a microbiology request stating "?infection" or "shunt infection" with a corroborating record entry where available.

Equivocal cases, such as mechanical revision in a patient with isolated, mildly raised inflammatory markers, were classified according to whether the operating team documented clinical suspicion of infection before or at the time of surgery. Where the microbiology request wording and the clinical record disagreed, the clinical record (operative note and concurrent electronic entries) took priority.

Data extraction and verification

A single author reviewed the available records and extracted data into a standardised spreadsheet. The same reviewer assigned sampling classifications and recorded outcomes. There was no independent second-reviewer assessment.

Outcome definitions

The primary outcome was a culture-driven change in management, which we defined as a documented action that the clinical record or microbiology advice attributed to the culture result, which was either starting, changing, or extending antimicrobial therapy; return to theatre, shunt removal, or externalisation; or readmission or re-operation for infection related to the result. Management changes were identified from the electronic patient records or documented microbiology advice or report comments. Decisions were not identified from any multidisciplinary team records.

Secondary outcomes were culture positivity, defined as growth of any organism from an eligible CSF specimen on direct or enrichment culture, and antibiotic initiation for any reason, including empirical therapy started on clinical grounds before culture results were available. Available microbiology comments on clinical significance were recorded as documented. For each positive culture, the organism and any microbiology comment on likely clinical significance were recorded.

Data analysis

Categorical variables were presented as counts and percentages. Given the descriptive nature of the audit, no statistical analysis or adjustments for confounding were performed. Device and procedure types were reported by sampling indication to show differences in case mix between the groups.

Unit of analysis and missing data

The primary unit of analysis was the individual specimen. Unique patients and the number of patients contributing more than one specimen were also recorded. Repeated specimens were retained because each represented a separate sampling decision. There were no missing data for sampling classification, culture result, or management-change variables.

Ethical considerations

The project was registered locally as a clinical audit (project no. 18462). Formal research ethics approval was not required for this retrospective service evaluation.

## Results

A total of 78 CSF specimens from 65 patients met the eligibility criteria, and 9 patients contributed more than one specimen. Routine (infection not suspected) sampling accounted for 60 of 78 specimens (76.9%), and clinically indicated (infection suspected) sampling accounted for 18 of 78 (23.1%), demonstrating that routine intraoperative sampling persisted despite first-cycle guidance. Specimen and procedure characteristics are summarised in Table 1.

**Table 1:** Cohort and procedure characteristics

Characteristic	Overall (n=78)	Routine (n=60)	Clinically indicated (n=18)
Device: ventriculoperitoneal shunt	62 (79.5%)	52 (86.7%)	10 (55.6%)
Device: lumboperitoneal shunt	8 (10.3%)	6 (10%)	2 (11.1%)
Device: ventriculoatrial shunt	1 (1.3%)	1 (1.7%)	0 (0.0%)
Device: external ventricular drain	6 (7.7%)	0 (0.0%)	6 (33.3%)
Device: subduroperitoneal shunt	1 (1.3%)	1 (1.7%)	0 (0.0%)
Procedure: insertion	50 (64.1%)	39 (65.0%)	11 (61.1%)
Procedure: revision	13 (16.7%)	12 (20.0%)	1 (5.6%)
Procedure: exploration	5 (6.4%)	4 (6.7%)	1 (5.6%)
Procedure: combined revision and exploration	6 (7.7%)	5 (8.3%)	1 (5.6%)
Procedure: removal	2 (2.6%)	0 (0.0%)	2 (11.1%)
Procedure: externalisation	2 (2.6%)	0 (0.0%)	2 (11.1%)

Culture yield by indication

Overall positivity was 23.1% (18/78). Positivity was higher among clinically indicated samples (8/18, 44.4%) than routine samples (10/60, 16.7%) (Table 2).

**Table 2:** Culture yield by sampling indication

Outcome	Routine (n=60)	Clinically indicated (n=18)	Overall (n=78)
Positive cultures, n (%)	10 (16.7%)	8 (44.4%)	18 (23.1%)
Negative cultures, n (%)	50 (83.3%)	10 (55.6%)	60 (76.9%)

Of the 10 routine positive cultures, 5 grew coagulase-negative staphylococci: three *Staphylococcus epidermidis*, one *Staphylococcus hominis*, and one *Staphylococcus haemolyticus*. The remaining five grew *Serratia marcescens *complex, *Micrococcus luteus*, *Acinetobacter radioresistens*, *Bacillus *species, and *Kocuria rhizophila*, with one specimen each. None of the routine positive cultures were associated with antibiotic initiation or a change in management. Out of the eight clinically indicated positive cultures, four grew *S. aureus*, three grew coagulase-negative staphylococci (two *S. epidermidis *and one *S. hominis*), and one grew *Enterococcus faecium*. Seven were associated with antibiotic initiation and a change in management; the remaining specimen grew *S. hominis*, was described as skin flora, and was associated with neither outcome. The *Serratia *isolate was identified in a patient undergoing a ventriculoperitoneal shunt for an unknown cause of hydrocephalus and returned to theatre later due to mechanical issues rather than infection, while the *Acinetobacter* isolate did not have any features of infection, and therefore neither of them was treated.

Clinical impact of positive cultures

No routine positive culture was associated with a documented change in management (0/10 routine positive cultures; 0/60 of all routine samples). Among clinically indicated positive cultures, 7/8 (87.5%) led to a culture-driven change in management. Across all 78 specimens, 7 (9.0%) led to a culture-driven management change, all in the clinically indicated group (Table 3).

**Table 3:** Culture-driven changes in management by sampling indication

Outcome	Routine (n=60)	Clinically indicated (n=18)	Overall (n=78)
Positive cultures	10	8	18
Culture-driven management change among positives, n/N (%)	0/10 (0%)	7/8 (87.5%)	7/18 (38.9%)
Culture-driven management change across all specimens, n/N (%)	0/60 (0%)	7/18 (38.9%)	7/78 (9.0%)

Antibiotic initiation

Antibiotics were started in 7 of 78 (9%) samples, all in the indicated group following a positive culture. No antibiotics were initiated in the routine sampling cohort or in the 10 culture-negative clinically indicated specimens.

Documentation consistency

Microbiology request wording and clinical adjudication disagreed on the indication in 14 of 78 specimens (17.9%). In five, the request implied infection, but the case was classified as routine. In nine, the request did not mention infection, but the case was classified as clinically indicated. This variability supports structured indication documentation.

Comparison with the first cycle

As the first cycle did not separate routine from indicated samples, a direct subgroup comparison or a claim of improvement between cycles could not be supported; the higher treated proportion in the second cycle reflects purposeful inclusion of infection-suspected cases rather than a change in treatment threshold (Table 4).

**Table 4:** Comparison of first and second audit cycles

Metric	First cycle	Second cycle
Total samples	172	78
Negative cultures	92.0%	76.9%
Positive cultures	8.0%	23.1%
Treated	4.0%	9.0% (all clinically indicated)
Routine versus clinically indicated split	Not recorded	Routine 76.9%, clinically indicated 23.1%

## Discussion

Routine intraoperative CSF sampling persisted in more than 75% of samples despite first-cycle guidance. Routine sampling produced positive cultures but did not change documented management (0/60), whereas positive cultures were more common when infection was suspected (44.4% vs. 16.7%) and accounted for every recorded change in management. All antibiotic initiation occurred in indicated cases, and documentation of indication was discordant in 17.9% of samples. These findings align with current evidence and guidelines that do not support routine CSF sampling for infection surveillance in diversion devices [2,11].

The finding that routine sampling produced positive cultures without changing any documented management (0/60) also coincides with the literature on surveillance CSF sampling, which consistently shows additional culture diagnoses without clinical benefit. Fernandez et al. evaluated effects of biweekly CSF screening with clinically indicated sampling in 562 EVD patients and found that screening approximately tripled culture-confirmed ventriculostomy-related infection diagnoses (OR 2.86, 95% CI 1.36-6.73) but did not change the proportion treated (OR 0.90, 95% CI 0.57-1.54). It also did not detect infection earlier or reduce permanent hydrocephalus, concluding that frequent sampling may itself contribute to infection [12].

Not all published data favour less sampling, and evidence on CSF sampling is mixed. Champey et al. reported a lower EVD-related infection rate (1.4%) at a centre performing daily CSF sampling compared to two other centres (7.2-9.2%). However, that centre also administered intraventricular antibiotics prior to drain removal along with their EVD care bundle. Therefore, the benefit cannot be attributed to sampling frequency and an accurate comparison cannot be made [13]. Acknowledging this outlier strengthens rather than weakens the case, as it shows us why a prospective, protocolised evaluation, rather than further retrospective audit, is required to establish the safety of an indication-based pathway. In our cohort, none of the 60 routine samples led to a change in management, and all treatment decisions followed clinically indicated sampling. The 17.9% documentation inconsistency also highlights the need to record sampling indications consistently, both to identify unnecessary testing and to ensure that genuine clinical suspicion is recognised.

The two groups also differed in case mix (see Table 1). All six EVD specimens were clinically indicated, while routine specimens mostly arose from shunt insertions and revisions. The higher culture positivity and treatment rates in the clinically indicated group therefore partly reflect some confounding by indication, rather than an effect of sampling itself. Patient characteristics, underlying diagnoses, and device type may also influence infection risk; however, they were not adjusted for.

The one clinically indicated positive culture that did not change management grew *Staphylococcus hominis*, reported as skin flora. Under IDSA criteria, a skin commensal isolated once, with normal CSF parameters and no fever, is more consistent with contamination than infection [2], and coagulase-negative staphylococci isolated from broth alone usually represent contamination [10]. Coagulase-negative staphylococci and other skin flora may represent both common contaminants and the most common pathogens [5,6], which explains why routine sampling generates clinically ambiguous results.

The small number of samples also limits precision. Having only 60 routine specimens and 10 routine positive cultures, with the absence of any culture-driven change in management, cannot exclude a small but clinically relevant rate of change in a larger cohort; furthermore, the proportion of clinically indicated cultures that changed management (87.5%) rests on eight cultures. Even though the findings are consistent between both audit cycles and published literature, their magnitude should be interpreted with caution. Finally, the absence of a documented change in management after routine positive cultures may reflect appropriate clinical interpretation of probable contaminants, but it does not, on its own, demonstrate that omitting routine sampling would be safe or without benefit. A comparison of this audit with the principal published studies of routine versus indicated sampling is summarised in Table 5.

**Table 5:** Present audit in the context of published studies of routine versus clinically indicated CSF sampling CSF: cerebrospinal fluid; EVD: external ventricular drain

Study	Design and setting	Comparison	Key findings relevant to this audit
This audit (second cycle)	Retrospective census, single UK centre, 78 intraoperative specimens	Routine versus clinically indicated intraoperative sampling	0/10 routine positive cultures (0/60 routine specimens) changed management; all seven culture-driven changes followed clinically indicated sampling
Steinbok et al. [9]	Retrospective, paediatric, 174 shunt revisions	Shunt-component cultures without CSF growth or clinical infection	19 cases of asymptomatic contamination; one treated; no excess malfunction
Fernandez et al. [12]	Retrospective, 562 EVD patients	Twice-weekly screening versus clinically indicated	Screening increased culture-confirmed infection (OR 2.86) but no change in proportion treated or outcomes
Williams et al. [14]	Prospective versus historical, two intensive care units, 383 patients	Daily versus every-third-day EVD sampling	Ventriculitis fell from 17% to 11% and proven ventriculitis from 10% to 3%; less frequent sampling (OR 0.44)
Jamjoom et al. [15]	Prospective, 21 UK and Ireland units, 495 EVDs	Sampling frequency and other risk factors	Daily sampling independently associated with infection (HR 4.73)
Champey et al. [13]	Retrospective, 3 French intensive care units, 462 patients	Daily sampling within a bundle including pre-removal intraventricular antibiotics versus two other centres	Infection 1.4% versus 9.2% and 7.2%; lower infection rate at the daily-sampling centre, but confounded by co-interventions
Palmiero and Figueiredo [11]	Systematic review, 14 studies of EVDs	Routine versus clinically indicated sampling	Only two direct comparisons; neither showed a benefit of routine sampling

Limitations

Several limitations qualify study findings. First, this was a retrospective audit at a single tertiary centre, so the results may not apply to units with different patient populations, surgical protocols, or microbiology practice. Second, both the sampling indication and the management changes were determined from clinical records, operative notes, and microbiology request wording, so incomplete or inconsistent documentation may have caused misclassification of either variable. The 17.9% discrepancy between request wording and clinical adjudication highlights the limitation of relying on documentation. Third, a single author classified every specimen and ascertained every outcome from the same records and was not blinded to the audit question. Inter-rater reliability therefore could not be assessed, and classification bias cannot be excluded, especially for judgement-based categories such as clinical suspicion of infection, where differences in clinical judgement and documentation may influence which specimens are sent and how they are classified. Fourth, repeated specimens from nine patients were analysed as separate sampling episodes. Although each represented a distinct decision point, these patients may have disproportionately influenced the findings. Fifth, the numbers of positive cultures (n=18) and clinically indicated specimens (n=18) were small, and no formal statistical testing was undertaken, consistent with the descriptive nature of an audit, so the estimates should be interpreted as hypothesis-generating. Sixth, no standardised, predefined infection definition linking culture results to CSF cell count, glucose, and protein was applied. Some positives could therefore not always be formally classified against IDSA contamination, colonisation, or infection categories [2]. Seventh, follow-up was limited to the local electronic records during the audit period and therefore, late infections, or infections managed at other hospitals, may have been missed. Critically, the absence of documented harm in a retrospective dataset cannot establish the safety of reducing routine sampling; this can only be determined prospectively, with a predefined missed-infection rate as a balancing measure.

## Conclusions

Across two audit cycles, routine intraoperative cerebrospinal fluid sampling during diversion procedures was low-yield and prone to contamination and did not change documented management, whereas clinically indicated sampling identified the infections that were clinically relevant and changed management. Routine sampling nonetheless persisted after the first cycle, and documentation of the sampling indication was often inconsistent. These cycles support reviewing the practice of routine intraoperative sampling in elective diversion surgery, introducing a mandatory structured indication field in operative documentation, aligning microbiology request wording with the operative indication, and providing targeted education for surgical and theatre teams.

Because of the small number of events and the retrospective design, these findings do not show that reducing routine sampling is safe. They justify a prospective evaluation of a documented indication-based pathway, with the missed-infection rate as a predefined balancing measure, followed by a further audit cycle to confirm that any reduction in routine sampling does not compromise the detection of genuine infection.

## References

[1] Ramasy Razafindratovo RM, Migliavaca CB, Chevret S, Champeaux-Depond C (2023). Internal ventricular cerebrospinal fluid shunt for adult hydrocephalus: a systematic review and meta-analysis of the infection rate. Neurosurgery.

[2] Tunkel AR, Hasbun R, Bhimraj A (2017). 2017 Infectious Diseases Society of America's clinical practice guidelines for healthcare-associated ventriculitis and meningitis. Clin Infect Dis.

[3] Choux M, Genitori L, Lang D, Lena G (1992). Shunt implantation: reducing the incidence of shunt infection. J Neurosurg.

[4] Pierce G, Thomas L, Peter P (1985). Infections of central nervous system shunts. Med Clin North Am.

[5] Conen A, Walti LN, Merlo A, Fluckiger U, Battegay M, Trampuz A (2008). Characteristics and treatment outcome of cerebrospinal fluid shunt-associated infections in adults: a retrospective analysis over an 11-year period. Clin Infect Dis.

[6] Claunch KM, DePerrior S, Rajnik M, Adams DJ (2026). Microbiology and epidemiology of pediatric and young adult ventriculoperitoneal shunt infections. Clin Pediatr (Phila).

[7] Fernández-Méndez R, Richards HK, Seeley HM, Pickard JD, Joannides AJ (2019). Current epidemiology of cerebrospinal fluid shunt surgery in the UK and Ireland (2004-2013). J Neurol Neurosurg Psychiatry.

[8] Koschnitzky JE, Yap E, Zhang Y (2023). Inpatient healthcare burden and variables influencing hydrocephalus-related admissions across the lifespan. J Neurosurg.

[9] Steinbok P, Cochrane DD, Kestle JR (1996). The significance of bacteriologically positive ventriculoperitoneal shunt components in the absence of other signs of shunt infection. J Neurosurg.

[10] Meredith FT, Phillips HK, Reller LB (1997). Clinical utility of broth cultures of cerebrospinal fluid from patients at risk for shunt infections. J Clin Microbiol.

[11] de Oliveira Manduca Palmiero H, Gadelha Figueiredo E (2026). Routine versus clinically indicated cerebrospinal fluid sampling for infection in external ventricular drains: a systematic review. Neurol Sci.

[12] Fernandez A, Uvelius E, Kronvall E (2024). Ventriculostomy-related infections: reduced incidence by terminating cerebrospinal fluid screening. World Neurosurg.

[13] Champey J, Mourey C, Francony G (2019). Strategies to reduce external ventricular drain-related infections: a multicenter retrospective study. J Neurosurg.

[14] Williams TA, Leslie GD, Dobb GJ, Roberts B, van Heerden PV (2011). Decrease in proven ventriculitis by reducing the frequency of cerebrospinal fluid sampling from extraventricular drains. J Neurosurg.

[15] Jamjoom AA, Joannides AJ, Poon MT (2018). Prospective, multicentre study of external ventricular drainage-related infections in the UK and Ireland. J Neurol Neurosurg Psychiatry.

